# Organizational Forms and Welfare Coalitions: Corporate Law and the Movement for Social Insurance in the US and UK

**DOI:** 10.1111/1468-4446.70041

**Published:** 2025-10-17

**Authors:** Maya Adereth

**Affiliations:** ^1^ London School of Economics and Political Science London UK

**Keywords:** Comparative politics, historical sociology, institutional political economy, law, trade unions, welfare state

## Abstract

Scholars of the welfare state have long argued that, in liberal democracies, welfare state expansion depends on successful coalitions in its favour. Under what circumstances do these coalitions form? Party systems, economic interest, and political mobilisation have all been thought to influence the emergence of coalitions for welfare state expansion. In this article, I argue that law plays a critical role in facilitating the last of these factors. Drawing on a growing body of literature that sees law as constitutive of, rather than merely reflective of, social relations, I demonstrate that available legal forms meaningfully inform opportunities for welfare coalitions. In particular, I examine how debates over what a trade union is—a voluntary association of individuals, or a corporate body deserving of a state statute—shaped coalitions for welfare reform in the US and the UK at the turn of the twentieth century.

## 1 Introduction

Across the literature on welfare states, coalitions in support of benefits are recognized as necessary, if not sufficient, conditions for their successful expansion. What enables these coalitions to form and endure? An institutionalist view suggests that electoral systems shape coalitions for redistribution—with proportional representation systems more likely to facilitate such coalitions than majoritarian ones (Iversen and Soskice 2006). Economically oriented accounts instead emphasise shared labour market risks or profitability concerns that variously align different classes, occupations, and sectors in favour of state benefits (Esping‐Andersen 1990; Hall and Soskice 2001; Korpi 2005; Swenson 1997). Finally, politically driven accounts highlight the importance of active organization, mobilization, and narrative framing by actors or groups (Esping‐Andersen 2017; Mann 1973; Przeworski 1977).

In what follows, I argue that law is an important yet underexplored avenue for understanding the conditions for welfare state coalitions. While a growing body of literature demonstrates how legal forms have shaped the evolution of business practices, far less attention has been paid to their impact on the other side of this arrangement—movements for social protection. Both this strand of legal scholarship and the literature on welfare coalitions thus benefit from this dialogue.

My argument is rooted in a historical comparison: throughout the nineteenth century, organized labour movements in both the United Kingdom and United States actively opposed government social insurance provision, instead cultivating their own expansive system of voluntary benefits (Adereth 2024). In both countries, the early twentieth century saw the rise of an influential group of elite reformers who actively campaigned to expand the role of government in the economy through a range of social policies. Couching their proposals in a language of national strength and social harmony, these reformers saw poverty as an obstacle to the fulfillment of a free society. The movements' respective platforms were both structured around the creation of a ‘minimum standard of life’ through the expansion of educational opportunities, an extensive system of social welfare, and the democratisation of economic relations. But they differed in one crucial respect: their relationship to organised labour. Whereas British reformers successfully recruited the Trades Union Congress (TUC) into their campaigns for state insurance, American Progressives were unable to forge a similar coalition with the American Federation of Labour (AFL).

The division would be consequential: in the postwar period, the UK launched the most extensive and inclusive national health system in the industrialized world. Already in 1911, a coalition between British trade unions and Liberal reformers had successfully passed state benefits that would form the basis for the country's expanded postwar welfare state. In contrast, the US saw no such achievements: it uniquely finished World War I without a single success in the introduction of state‐ or federal‐level benefits, leaving it poorly positioned to enact welfare reforms in the postwar period.

Here were two majoritarian electoral systems experiencing similar economic shocks due to rapid industrialization. In both cases, a group of elite reformers would seek to respond to these shocks through forging a greater role for the state in managing economy and society. Trade unions legalisation, in both reform movements, would be advocated as a means for integrating organized labour into the administrative machinery of the state. If allied with government, reformers argued, trade unions could go far in raising standards of living, managing industrial disputes, and regulating prices. In doing so, legal recognition would serve a public good that would smooth over class conflict.

Though they shared similar objectives, reformers in each country were faced with different legal categories for the advancement of such legitimation. Since the early 19^th^ century, British workers associations for mutual benefit (friendly societies) had been granted a special status of ‘quasi‐corporation.’ In exchange for registration, this status enabled such societies to hold assets, make binding contracts, and appear in court through property owning trustees agreed upon by membership and government. With no corporate charter, however, they could not be sued in court for the actions of their members. In the US, workers' associations viewed as legitimate by governing elites had, since the late 18^th^ century, been directly issued corporate charters. These charters enabled ‘benign’ associations to make legally binding contracts, accumulate funds, appear in court, and own assets. But they also subjected these corporate entities to direct state supervision over their bylaws, internal documents, and financial accounts. Even more concerningly, they rendered such associations liable to be sued in court. Because of the direct state supervision and legal liability it entailed, full incorporation became increasingly objectionable to organised labour by the end of the nineteenth century.

In both cases, reformers viewed legal recognition as a path to greater respectability. A clear legal status, they argued, would tame trade unions and enable them to work alongside government and employers in negotiating adequate conditions of work and pay—reducing the threat of wildcat strikes and leading to cooperation over conflict. In both cases, organised labour viewed full incorporation as an excessive infringement on its capacity to effectively represent workers. The legal forms available for categorizing trade unions offered a crucial medium for navigating these interests—one which shaped coalitional prospects between reformers and labour leaders in each case.

In the national debates that took place in 1867, 1894, and 1901, British reformers advocated for the expansion of the quasi‐corporate status to trade unions—thereby increasing their respectability without alienating labour leaders. In the US, reformers pursued the only avenue of legitimation they had available: that of full incorporation. American Progressives actively campaigned for trade union incorporation, arguing that it would grant trade unions a seat at the bargaining table and integrate labour into the country's industrial relations infrastructure. Their proposals culminated in the Hepburn Bill of 1908, a proposal to legalise trade unions in exchange for their registration with the Bureau of Corporations. The proposal was immediately opposed by labour leaders across the board, who desperately wanted legalisation but absolutely rejected that it should come in the form of incorporation. Constant legal proceedings, they feared, would deplete their funds and drive them towards extinction.

Though both groups of reformers sought close collaboration with organised labour, the legal avenues through which such collaboration could be sought impacted the fate of the two attempted coalitions. In repeated debates on the question of trade union incorporation, UK labour leaders came to trust that Liberal Reformers sought the protection of their unions. American labour leaders, by contrast, grew increasingly suspicious of the Progressive movement. Whereas the TUC joined Liberal Party reformers in advancing early social insurance proposals, the AFL grew overtly hostile to Progressives and subsequently distanced itself from their campaigns for state aid. The legal forms available to welfare advocates, and their resulting position on the question of trade union incorporation, would meaningfully shape coalitions for welfare reform**.**

### 1.1 Law and the Making of Welfare Coalitions

Three strands of thinking have most notably characterized theories of support for the welfare state. The first, institutional strand, attributes deviations in support for welfare state expansion a country's electoral system. Whereas proportional representation systems are thought to promote centre‐left parties prone to redistribution, majoritarian systems tend to produce centre‐right governments less likely to redistribute (Iversen and Soskice 2006). Disaggregated federal institutions, similarly, are thought to weaken class‐based mobilization and fracture work‐based welfare coalitions (Hacker 1998; Skocpol 1992).

Yet another set of explanations focus on economic interest. Despite placing emphasis on different economic actors, both the Power Resource and Varieties of Capitalism schools of welfare scholarship highlight market or life risks as a core component of redistributive coalitions—whether between farmers, the middle class, and the working class, or between employers and workers (cite). To become politically relevant, however, these interests must be formulated by interested political actors. Parties, trade unions, and other organizations have, at least since Antonio Gramsci, been recognized as active agents in the formulation and articulation of shared political interest. Rather than appearing as objective truths, a number of authors have suggested that shared political interests need to be actively constructed and solidified in the course of collective struggle (Esping‐Andersen 2017; Mann 1973; Przeworski 1977).

The capacity to construct these coalitions, however, is not without its own constraints. In particular, legal forms and categories inherited by political movements have the potential to fundamentally shape relations within them. As argued by early institutional economists like JR Commons and Thorstein Veblen, the law is crucial to social relations because it deliberates between “two wills at a given point in time” (Commons 1924:79). An expansive body of literature has considered how the influence of the courts, the persistence of the master‐servant laws, and laws of conspiracy have been used to undermine the power of organized labour in the United States (Ernst 1995; Forbath 1991; Hattam 1993; Orren 1991; Tomlins 1985). The role of legal organizational categories, however, has remained unexplored.

The importance of legal organizational categories *has* been embraced by scholars like Katharina Pistor and Brooke Harrington, who have demonstrated how the legal categories of trust and corporation actively shaped the evolution of industrial capitalism (Harrington 2017; Pistor 2020). But this work has thus far not been applied to other economic actors. Insofar as the specific attributes of capitalism vary with diverse linkages between associations of firms, workers, civil society and government institutions, understanding capitalist development necessitates paying equal attention to the organisation of labour as to the organisation of firms. Just as legal forms can cloak assets with specific rights and limitations, they shroud all social activity in a web of characteristics which influence their character and historical behaviour. While the literature on welfare coalitions stands to benefit from closer consideration of legal forms, then, work in the tradition of law and political economy (LPE) can equally benefit from examining the implications of law for distributional politics.

### 1.2 Organizational Law and Workers Associations

During the late 18th and 19^th^ centuries, the UK and US constituted two exemplary “associational states.” In both countries, governments promoted freedom of association and civic engagement through clubs and societies. They also, however, carefully managed collective action by organized groups. Associations in the two countries took three primary forms: the first, and least regulated, were voluntary associations. These were soluble associations of individuals which could form and disintegrate with little government oversight. The organisations, in other words, had no legal status independent from that of the individuals who composed them. Voluntary associations benefited from limitations on the degree of state intervention and oversight they received—with no obligation to share their internal accounts, administrative records, and by‐laws. On the other hand, the contracts they formed with their members had the same legal status as agreements between individuals, and thus were difficult to enforce in court. Moreover, as combinations of individuals, voluntary associations were susceptible to accusations of conspiracy in restraint of trade.

On the other end of the spectrum were corporations. At their core, corporate entities are unique in their “ability to own property, enter into contracts, and stand in court independently of [their] owners” (Dari‐Mattiacci et al. 2017, 194). Among their most definitive attributes is perpetual succession—unlike partnerships which depend on the commitment of specific shareholders to survive, or trusts whose existence depends on the decisions of their beneficiaries, corporations are everlasting. They are “invisible, immortal, and rest only in intendment and consideration of law” (Holdsworth 1922, 386). Because they facilitate long term capital commitments, corporations have been intimately linked with the evolution of industrial capitalism and the emergence of modern states (Dari‐Mattiacci et al. 2017; North and Weingast 1989; Zhang and Morley 2022).

**Table:** 

	Permanent and independent legal identity?	State access to financial accounts and internal documents?	Legal standing of contracts?	Liable to suit?	Ability to own assets?
Voluntary association	N	N	N	N	N
Corporation	Y	Y	Y	Y	Y

In both the US and the UK, voluntary associations and corporations formed the organizational pillars of state formation, ones which helped mediate between an ostensible commitment to protecting the rights of individuals and a powerful suspicion of organised groups. Voluntary associations were representative of the prized sphere of civil society, one which faced little state intervention but also held no legal rights or capacities for resource accumulation. Corporations were the legal recognition of organised groups—ones which benefitted from legal rights at the cost of heavy state supervision and liability to suit. The prolific Chicago attorney William Niblack observed that “While a society remains unincorporated it may make such rules and regulations as may seem proper for the discipline of its members, but as soon as it becomes incorporated it surrenders this power and becomes subject to the visitorial power of the courts” (Niblack 1894, 45). Characteristic of the corporate form is therefore a tradeoff between recognition and subordination.

Some of the first corporations in British history were the guilds of the Weavers and Goldsmiths, Fishmongers, and Merchant Tailors, who obtained Royal Charters of Incorporation in 1327, 1433, and 1466 respectively (Williston 1888). In the mid‐17^th^ century, Royal Charters would be granted to municipalities, towns, guilds, and universities as legal recognition in exchange for loyalty to the Crown. In the US, as in the UK, incorporation served to supplement limited state capacity and was initially granted through special acts of legislature (Zhang and Morley 2022). Histories of the early American state suggest that incorporation enabled a sort of inconspicuous expansion whereby the American state could control and manage various parts of society without growing its bureaucratic institutions. In the aftermath of the Civil War, corporate charters allowed the overcoming of opposing sectional interests and the management of decentralised pressure groups. This was facilitated by the requirement that corporations serve a fundamental “public purpose.” Collaboration with private actors lent government a subtle infrastructural power which offered stability while allowing for dynamism—encouraging collective action without the sense of state coercion (Clemens 2020).

The corporation was thus a private entity deeply intertwined with the public sector. In the words of William Novak, corporations were “legal and political constructions rather than spontaneous private collaborations” (Novak 2001, 163). 19th century legislators, judges, and commentators depicted corporations as constitutive components of the legal‐constitutional state—delegations of rule‐making authority and public resources. They therefore represented an active exercise of public power—in the words of the jurist Francis Lieber, they constituted "a vast system of institutions whose number supports the whole as many pillars support the rotunda of our capital” (Novak 2001, 174).

## 2 Legal Forms and Organised Groups: Diverging Trajectories

Despite their apparent similarities, the legal categories of voluntary associations and corporations in the US and UK in practice had diverging political trajectories. Until the Joint Stock Companies Act of 1844, British corporations were exclusively formed through special Royal Charters or, after 1688, directly by Parliament. This greatly limited the number of corporations formed—the process depended not only on status and funds, but on the alignment of the organisation's interest with those of the state. After the economic collapse of the South Sea Bubble, the Bubble Act of 1720 outlawed all private companies except those holding a corporate charter (Holdsworth 1922; Williston 1888). But absent general incorporation laws, the issuing of a corporate charter remained costly and time intensive.

It was thus difficult to extend complete corporate rights to workers associations. After the repeal of the Combination Acts and the Bubble Act in 1825, however, the English government increasingly sought to extend some sort of legal recognition to groups of workers organized for benign purposes. That year, they developed a “quasi‐corporate” status used to regulate the popular and proliferating mutual benefit or “friendly” societies. As a recognition of their utility in promoting working‐class thrift, friendly societies received protection from the government through legal enforcement of their contracts and by‐laws, as well as the right to hold property, sue or be sued through appointed trustees. Though their rules and records had to be submitted for review by a registrar, the latter could not interfere in their formulation. In this way, the societies gained some freedoms of voluntary associations and some parliamentary protections characteristic of corporations. They could also effectively act like corporations through trustees (House of Commons 1825).

**Table:** 

	Permanent and independent legal identity?	State access to financial accounts and internal documents?	Legal standing of contracts?	Liable to suit?	Ability to own assets?
Quasi‐corporation	N	Highly limited; no intervention	Y Via trustee	N	Y Via trustee

Friendly societies preserved their ambiguous legal form throughout the nineteenth century, exempt from regulations on joint‐stock companies, insurance companies, and other corporate bodies while benefitting from legal enforcement of their contracts, the ability to accumulate funds and stand in court.

No such middle‐ground was forged in the US, where general incorporation was standardized and widely available. By the early 19th century, the rate of incorporation took on immense new proportions—states like Massachusetts, New York and Pennsylvania enabled general incorporation by registration as early as 1789, 1791, and 1787 respectively. The US produced almost 350 corporations between 1783 and 1801 (Arner 2002). By 1790, Massachusetts alone had passed 200 acts of incorporation.

During the 1830s, American states had gained the capacity to credibly regulate and enforce corporate statutes, and general incorporation laws proliferated—with 27 out of 32 states adopting general incorporation acts by 1860. Notably, few of these corporate entities conducted business for profit. Rather, the overwhelming majority were chartered for communal reasons as municipal, charitable, or educational organisations (Zhang and Morley 2022). Contemporary observers remarked that in the US “charters of incorporation for mere economical purposes, as the construction of roads and canals…were recognised to be more frequent… than in any other country” (Maier 1993, 51).

Associations “for any literary, charitable, or religious purpose” could easily file for a corporate charter (Lamoreaux and Novak 2017). Early benefit societies in the US thus gained recognition through statutes of incorporation. In 1848, NY allowed for the incorporation of “benevolent, charitable, scientific, and missionary societies.” In the following 2 decades, California, Ohio, Maryland, North Carolina, New Jersey, Kentucky, Massachusetts, Kansas, Iowa Illinois, and Wisconsin passed similar regulations (Bloch and Lamoreaux 2015). Incorporated benefit societies were viewed as a means to provide a legitimate alternative to “wasteful” working‐class leisure activities, encouraging savings and thrift as a means of preventing the rise of political radicalism (Bloch and Lamoreaux 2015). Between 1789 and 1865 the state of Connecticut granted 3000 "special" acts incorporating and regulating benefit associations, while the state of Georgia incorporated more than 50 mutual benefit societies, compared to 64 manufacturing firms. This was typical of other states including Illinois, Massachusetts, Maine, North Caroline, and Tennessee (Novak 2001).

### 2.1 Trade Unions: Between voluntary associations, corporations, and Quasi‐Corporations

Where did trade unions fit in this legal landscape? By the early twentieth century, it was difficult to equate the national amalgamated trade union federations to neighbourhood book clubs or debating societies. In the US, trade union membership ballooned from less than half a million in 1897 to more than two million in 1904. In the UK, trade unions reached two million members in 1900 (Wolman 1937). With coordination at the municipal, regional, and national level, benefit funds consisting of several million dollars, and infiltration across industries and sectors, late nineteenth century trade unions had become national political forces to be reckoned with (Commissioner of Labor 1908). A new legal framework outlining their rights and responsibilities became increasingly necessary.

In substance, the spectrum of responses to the rise of organised labour were remarkably similar in the two countries: on the one hand, employer‐backed conservatives sought to severely constrain or entirely do away with organisations of workers; on the other end, labour leaders sought to obtain legal rights for collective action and powerfully opposed trade union incorporation. Mediating between these was a group of elite reformers who sought to legalise trade unions in order to pacify them.

In his 1878 book, the Liberal British labour leader George Howell argued that trade unions

wield a vast power, socially and politically, and whether for good or for evil, it is daily increasing…It is useless to abuse them, to put them down is impossible; the only sensible way of dealing with them has been done by legally enfranchising them.

(Howell 1878: xxii)

Soon after, a seminal article by Liberal politician L.A. Atherley‐Jones would shake the party with its urgent call for collaboration with organised labour (Atherley‐Jones 1889). This collaboration would be oriented towards eliminating class conflict rather than provoking it. The Liberal sociologist L.T. Hobhouse presented trade unions as “arbiters of industry by the deliberate and systematic arrangement of labour and commerce in the best interests of society as a whole” (Hobhouse 1912, 46). Throughout the crisis of the 1890s, Liberal politicians increasingly encouraged collaboration in the name of the public good, advocating government managed conciliation schemes between two legally recognised actors (Powell 1986).

Similar arguments were echoed in the United States. Among the foremost inspirations for the Progressive movement was a 1909 book by the political philosopher Herbert Croly titled *The Promise of American Life*. This widely circulated book argued that

The labor unions deserve to be favored, because they are the most effective machinery which has as yet been forged for the economic and social amelioration of the laboring class. They have helped to raise the standard of living, to mitigate the rigors of competition among individual laborers, and in this way to secure for labor a larger share of the total industrial product.

(Croly 2014 [1909]: 387)

Inspired by Croly, the 1912 programme of the short‐lived Progressive Party, headed by former US President Theodore Roosevelt, called for the “organisation of workers—men and women—as a means of protecting their interests and promoting their progress” (Progressive 1912). Taking inspiration from European legislators, American Progressives called for “cooperation between the government and trades unions” through “orderly legislation” (Addams 1912, 4).

What differed between the two cases were not the *substantive intentions* of reformers but rather the *legal form* in which these intentions manifested. In the UK, trade unions could conceivably take the form of corporations or quasi‐corporations. With conservatives advocating for full incorporation, Liberal reformers could present the quasi‐corporate form as a reasonable alternative—one which had been used to tame threatening working class associations since the early nineteenth century. Indeed, in debates on the legal rights of trade unions, British reformers and trade union leaders joined forces in advocating the expansion of the quasi‐corporate status created for friendly societies to associations of workers for collective bargaining. In 1867, 1894, and in the aftermath of Taff Vale, reformers defended trade unions from full incorporation, which would entail intense regulation and open the possibility for more frequent prosecution. Their defence of trade unions' quasi‐corporate status would facilitate a repertoire of trust which contributed towards their long‐term coalition with organised labour.

Reformers in the US had one alternative to the category of voluntary association—the corporation. In the US context, it was the corporate form that had historically been used to recognise, regulate and pacify associations of citizens. In exchange for a legal standing, societies were required to compile and file a complete report of their financial accounts, communications with members, rules of conduct, and contractual agreements. By the end of the nineteenth century, such regulations had been implemented in New York, Massachusetts, Minnesota, Iowa, Michigan, Wisconsin, and the District of Columbia (United States Congress 1897, 630).

For much of the nineteenth century, rights of incorporations were explicitly denied to trade unions. More than half of the charters issued between 1807 and 1818 in New York specifically outlawed rules regarding wage rates, and nearly every corporate charter granted to a labour group before 1820 stipulated severe punishments for any unapproved objectives. 19th century legislators and judges extended corporate rights exclusively to voluntary associations that were regarded as politically neutral or benign, denying the opportunity to radical reform associations, anti‐slavery groups, and immigrant organisations in addition to trade unions with non‐benevolent ends (Bloch and Lamoreaux 2015). Initially, then, US trade unions actively sought the right to incorporate. The platform of the FOTLU set up in 1881 had "the passage of laws in state legislatures and in congress for the incorporation of trade unions and similar labour organisations" as its foremost aim (Kirk 1906, 622). Similar demands were made by the New York Workingmen's Assembly and the Knights of Labour (Committee on Labor 1886, 1; The Labor Publishing Company, 1886, 30). In testimonies before the Senate Committee on Education and Labour, subjects stress the desire to be “permitted to legalise our trade societies by incorporating them so that they may have the privileges and responsibilities of other corporate bodies” (Committee on the Relations Between Labour and Capital 1885:91).

In 1886, the O‐Neill Act granted trade unions the right to incorporate in the district of Columbia. Similar acts were passed in Maryland in 1884, Michigan in 1885, Iowa in 1886, Massachusetts in 1888, Pennsylvania in 1889, and both Louisiana and New York in 1890 (Ernst 1995; Hattam 1993). Trade unions across the board began to incorporate their insurance departments or modify their rules in line with state regulations for fraternal societies. During the late nineteenth century, then, incorporation presented a crucial, if limited, avenue for trade union legalisation.

But with rising injunctions in the late 19th century, labour leaders grew wary of the advantages of incorporation. In 1898, AFL leader Samuel Gompers declared that incorporation was “Inimical to the interest of labour and destructive of labour organisations,” noting, “When the whole history of jurisprudence has been against the laborer it would be foolhardy for organised labour to make itself more amenable to suit” (Ernst 1995, 155). An article from the Wall Street Journal quotes Gompers declaring that with incorporation, “The unions attacked from all sides would be in constant litigation” (The Wall Street Journal 1903, 1). The position was shared by other labour leaders. President of the United Mine Workers Union of America John Mitchell equally expressed his opposition (The New York Times 1903, 9). And in an interview, Cigarmaker's Union leader Adolph Strasser stressed, “at the present time I would advise no trade union to incorporate, because they cannot expect justice at the hands of the court… when a judge is against you by education or surroundings, he will always find a reason and argument to confirm him in his decision” (Marett 1904, 376).

Opposition to incorporation across the labour movement placed it starkly at odds with Progressive reformers, who persistently advocated for incorporation as the primary means of legal legitimation. As Herbert Croly himself argued, trade union recognition “must depend upon conformity to another set of conditions, imposed in the interest of efficiency and individual economic independence. In this respect [they] will be treated precisely as large corporations are treated” (Croly 2014 [1909]: 394). With the Hepburn Bill of 1908, Progressives proposed to offer trade unions a permanent legal standing in exchange for registration with the Bureau of Corporations. Fraught negotiations over the Bill would meaningfully erode relations between labour leaders and key figures of the movement for social insurance.

## 3 The Quasi‐relations Corporation and the Lib‐Lab Coalition

In the UK, the debate over trade union incorporation took place in three acts. In each instance, a conservative political faction attempted to force trade union incorporation. Liberal reformers defended against these attempts, pushing instead for the expansion of the quasi‐corporate status and forging ties with labour leaders in the process.

### 3.1 Hornby versus Close

The issue was first explicitly debated in the aftermath of the Hornby v. Close case of 1867. The case came out of a dispute between the United Society of Boilermakers and one of its members, which the society took to court for lack of dues payment. Formally registered as a friendly society, the Boilermakers theoretically had the backing of the state to enforce by‐laws and obligations on their membership. Upon review of their funds, which had increasingly been devoted to strike support, however, the judge determined that the society was organised for illegal purposes and thus not covered by friendly society law.

The case, alongside a wave of violent industrial disputes known as the Sheffield Outrages, prompted a Royal Commission investigation into the organisation and rules of trade unions. The Commission was initiated by the Conservative government with a view towards developing a fresh set of laws governing trade union organisation. During 2 years of investigations, national amalgamated societies like the Engineers, the Carpenters, and the Bricklayers systematically campaigned for legislation protecting trade unions under the same framework received by friendly societies (Klarman 1989).

The Commission's final report concluded that trade unions held two distinct functions: first those of a friendly or benefit society, and second those of a “trade society proper,” including the promotion of working‐class interests in key trades and the protection of workers from the disproportionate power held by employers. It was found that “the effect of the established societies [was] to diminish the frequency and certainly the disorder of strikes, and to guarantee a regularity of wages and hours” (UK Parliament 1868:xiv).

Though it acknowledged the legitimacy of trade union objectives other than the provision of mutual benefits, recognition of these alternative functions came with a call for greater regulation. The Commission's Conservative majority report concluded that “facilities should be granted for such registration as will give to the unions capacity for rights and duties resembling in some degree that of corporations, and to the public the means of knowing the rules, members, and funds of the unions, and also their expenditures and proceedings” (UK Parliament 1868:xxiv). Upon the granting of a corporate charter, the majority recommendation also implied that a trade union was liable to be sued for the actions of its members.

But already in this first debate, trade unions found willing allies. The minority report of the same Commission argued that, like friendly societies, trade unions “should acquire a right of registration under no condition other than submitting their rules to the registrar and satisfying him that they are not criminal.” Instead of corporations, the dissent argued “That every such lawful association be capable of obtaining the benefit of those parts of the friendly societies act” (UK Parliament 1868:xxxi). Just as friendly societies were granted independence from the state because of their promotion of self‐help and thrift, the minority held that trade unions deserved a degree of independence due to their promotion of industrial peace. Moreover, forced incorporation might actively prevent cooperative relations:

The amount of feeling which this question arouses on both sides, the great irritation of those who have suffered by trades unions, and the extreme jealousy on the part of their members of State interference, would, we are convinced, render the attempt to pass such a measure impracticable. We are far from seeing any certainty that such an Act is even ultimately desirable. Trades unions are essentially clubs and not trading companies, and we think that the degree of regulation possible in the case of the latter is not possible in the case of the former.

(UK Parliament 1868:lix)

Despite their weak ties to the ruling Conservative majority, labour leaders were effective in rallying support for the minority view from reformers both within and outside parliament (Klarman 1989). In an 1869 parliamentary debate, social reformer Thomas Hughs favourably observed that “On the whole the Trades Unions had been much better conducted of late than they had been for many years before. What they now asked was to be put upon the same footing as other societies before the criminal laws of the country” (UK Parliament 1869). Hughs argues that the outlawing of trade unions meant that the “numbers of secret societies sprang up,” leading to the “burning of mills, the destruction of machinery, the vitriol‐throwing, and the hangings which disgraced that period of our industrial history”(UK Parliament 1869). Just as the Friendly Societies Act encouraged “the best of those societies” to register with the state, so trade unions should be granted a similar status.

The Trade Union Act of 1871 granted trade unions “remedies similar to those of friendly societies”: that is, government enforcement of contracts, the ability to hold property and appear in court through trustees, and highly limited government supervision over by‐laws, communications, and financial accounts. The Act “free[d] trade unions from the last remains of their former character of criminal conspiracies” but denied them explicit rights against their members and prevented them from entering legally binding contracts as entities in their own right (UK Parliament 1871).

### 3.2 the 1894 Report

The idea that trade unions should incorporate to secure their regulatory features would, however, persist as industrial disputes rose. In 1891, the Salisbury Conservative Government appointed a Commission on Labour to assess the “leading causes” of modern disputes between workers and their employers, the institutions that may prevent them, and whether they might be reduced with government legislation. The commission, headed by economist Alfred Marshall, examined 583 witnesses over 151 sittings, producing “volumes of reports” on labour conditions across the empire and Europe. Beatrice Webb described it as a “symposium on the Labour question” (Groenewegen 1994, 276) which summarised a wide range of views on the legitimacy of trade unions and the right to strike (Groenewegen 1994).

Marshall's professional preoccupation was in the transformation of the country's workers into “gentlemen,” especially insofar as this would weaken the appeal of socialism. Under his guidance, the majority report advocate for “the extension of the liberty to bodies of workmen or employers to acquire fuller legal personality than that which they at present possess is desirable,” insisting that labour unions “should acquire some process of registration of a corporate character” in order to "afford, when both parties wish it, the means of securing the observance, at least for fixed periods, of the collective agreements which are not, as a matter of fact, made between them in so many cases.” It was held that the newly broadened “respectable” functions of trade unions—i.e. facilitating arbitration as an alternative to strikes—demanded a complete legal personality on both ends. Finding that “it is impossible to make strikes or lock‐outs illegal and punishable in any case… where a sudden strike in breach of contract may involve actual danger to the public,” the commission insisted that,

as things stand now, large bodies of workmen or employers cannot, as such, enter into legal contracts of submission to arbitration for want of legal personality and, for the same reason, damages cannot be recovered from them. If, however, the suggestions which we have made were adopted, and it were put within the power of such bodies to acquire legal personality sufficient to enable them to enter into collective agreements with the legal sanction of collective liability in damages for breach of such agreements, this difficulty would so far be solved.

(House of Commons 1894, 116–17)

The report acknowledged, however, that "The evidence does not show that public opinion is as yet ripe for the changes in the legal status of trades associations which we have suggested" (House of Commons 1894, 119).

Indeed, labour leaders expressed a powerful resistance to incorporation as put forward by the Commission. During the TUC Parliamentary Committee meeting of 1894, representatives note “the suggestion [of corporate powers] is one which will require to be carefully watched, otherwise alterations of an important character may be made in law relating to trade unions, the effect of which may completely change the constitution of these societies” (Trades Union Congress 1894, 24). At a speech before the Congress, Lib‐Lab MP Henry Broadhurst declared that

the act is calculated to impose grave obligations and to seriously imperil the position of trade unions of this country…I do hereby instruct the parliamentary committee to be alert in resisting any legislation of that character, as being utterly injurious to the true interests of trade unionism, and calculated to plunge us into costly and unnecessary litigation.

He resolved,

should any attempt be made in Parliament in this direction, no matter in what modified form or in what small degree, they should oppose it and summon the whole force of trade unionism throughout the United Kingdom to rally around them in opposition to the suggestion.

(The Times 1894, 10).

As in 1867, a cohesive and influential minority on the commission resisted these recommendations. Their report, compiled by trade union‐politicians like Michael Austin, William Abrahams, James Mawdsley, and Tom Mann, argued:

Any attempt to revoke [the Trade Union Act of 1871], or in any way to tamper with the purely voluntary character of their associations would, in our opinion, provoke the most embittered resistance from the whole body of trade unionists and would, we think, be undesirable from every point of view.

(House of Commons 1894, 146).

### 3.3 Taff Vale and the Trades Disputes Bill

This time, it was the majority view that would predominate. Among civil servants and the courts, a desire to integrate trade unions into processes of industrial arbitration provoked the conviction that it would be useful to offer trade unions the possibility of incorporation. As the civil servant Bertrand Holland argued, “the time has come to place trade associations upon the same footing that other large business partnership should before the law and to strike off the last disabilities imposed by the ancient jealousy of their action” (Holland 1895, 401).

The resonance of this position would soon become clear with the monumental decision on the Taff Vale Case of 1901. When the Taff Vale Railway Company sued the ASRS for damages perpetrated by its members during a strike, the judge held the union liable as a corporate entity. The decision stated that “if the Legislature had created a thing which can own property, which can employ servants, and which can inflict injury, it must be taken to have impliedly given the power to make it suable in a court of law for injuries purposely done by its authority and procurement" (UK Parliament 1902).

Taff Vale would spark a historic debate on the organisational character of trade unions and their attending rights. Supporters of trade union incorporation would ally with Justice Farwell in viewing the organisations as “creatures of statute” owing their legal validity to the Acts of 1871. By contrast, trade unionists inquired, “where in the trade union acts is it to be found any enactment, express or implied, that a trade union is to be sued in its registered name? Express there is none, and it is clear that a Trade Union is not made” (Trades Union Congress 1901, 31–32).

The Taff Vale decision prompted deeper reflection on the status of trade unions across the Liberal Party. At a Liberal Party meeting, the future Prime Minister H. H. Asquith held that “Liberals must be prepared to make greater concessions to the labour wing of the Liberal Party.” In the struggle over the question of incorporation, bonds between labour leaders and liberal reformers were formed and strengthened. In debates on the Trades Disputes Bill, a speech published by the Liberal Review holds, “the liberal government will find every encouragement in this report for acting boldly in rehabilitating the rights of trade unions, as far as those rights were assailed by the recent interpretation of offences” (The Liberal Review 1906, 494–95).

In the Daily Mail, it was reported that

Over 200 liberal and labour members have announced their intention of opposing any Bill which does not come up to the trade union standard as shown by their Bill already introduced. The more moderate Liberal members and an influential portion of the cabinet are equally determined that intimidation and immunity of all trade union funds shall not be legalised. There is a chance of serious friction over the Trades Disputes Bill in the near future.

(The Daily Mail 1906, 7).

Indeed, the TUC Parliamentary Committee insisted that “no Trade Disputes Bill will be satisfactory which does not secure what was understood as the ante‐Taff Vale position on the basis of the complete immunity of the funds of trade unions from litigation (Trades Union Congress 1906, 133).

The crucial Taff Vale decision, and subsequent Trades Disputes Bill, were fundamentally decisions on organisational form. In the former, the courts held that a trade union was equivalent to a corporation—an entity formed through the law and thus suable in its own right. Liberals and Progressives closely allied with trade unions to reverse the decision. In April of 1904 and March of 1905, Liberal MPs J. M. Paulton and T. P. Whittaker introduced bills to limit trade union liability which were supported by a majority of Liberal MPs. Both Asquith and Gladstone emphasised the importance of ensuring the “free power of effective combination which Parliament, after long and careful inquiry, has deliberately conferred” (Powell 1986, 385). In the 1906 elections, almost two thirds of Liberal candidates committed to alter trade union law in the latter's favour. The resulting alliance was essential to facilitating the launching of the Labour Party and the birth of Britain's skeletal welfare state.

## 4 Incorporation and the US's Failed Progressive Coalition

Less than two decades after the enactment of general incorporation statutes, incorporation had transformed from a demand by the labour movement into an imposition from outside. In the American Federationist, Samuel Gompers argued that with incorporation, employers “would make every effort ‘under the forms of law’ to mulct out unions in damages for supposed injurious results from trade union action” (Samuel Gompers, 1904:1086). A 1905 editorial by Gompers asks "would any fair‐minded court soberly maintain that a voluntary association called a union is different from a voluntary association called a club or a church?” reiterating that “for good and sufficient reasons…organized labour, with some exceptions, does not choose to assume the corporate status” (Samuel Gompers, 1905:631). And in an interview with JR Commons, JR Sullivan of the Topographical Union insists that “an unincorporated union is a law unto itself. It is free. An incorporated union would be subject to much revision and correction through law agencies…The knowing are fully conscious of what they are saying when they express a desire for an increase of the authority of the law over trade unions. They would wreck them from within” (Commons 1905, 139–40).

Opposition to incorporation would situate trade unions squarely against Progressive Reformers. In 1902 Commissioner of labour Carroll D Wright insisted that the question on trade unions was not whether they should exist, but how “to conserve their usefulness, to increase their responsibility, and to prevent their follies and aggressions.” The answer: “conferring upon them privileges enjoyed by corporations, with like proper restrictions and regulations” (Wright 1902). The division between labour leaders and reformers over the question of incorporation is perhaps best illustrated in a debate between Louis Brandeis and Samuel Gompers on the question. Brandeis begins,

[the] improvement in the condition of the workingmen has been almost a net profit to the community…And because the trade unions have accomplished much, and because their fundamental principle is noble, it is our duty where the unions misconduct themselves, not to attack the unions, not…to refuse to recognize them, but to attack the abuses to which the unions, in common with other human institutions, are subject, and with which they are afflicted…the incorporation of labor unions would in some measure tend to correct them…[would make them] more deliberate, less arbitrary, more patient with the trammels of a civilized community.

(Gompers and Brandeis 1998, 305).

Gompers responds mockingly,

Our friend says that this proposition to incorporate the trades unions ought to be welcomed by them. Well, we have not reached that stage of appreciation of this kind offer which is made to us (laughter.)…I protest against the attempt to invent new laws so as to create a new crime to apply to organized labor… when courts so far transgress upon the rights of wage earners, when they will invade the rights to which the toilers are entitled, you must excuse us, if you please, if we decline your invitation to step into your parlor (Loud applause).

(Gompers and Brandeis 1998, 311).

### 4.1 Loewe Versus Lawler and the Hepburn Bill

Tensions between elite reformers and organised labour over the question of incorporation would come to a head with the infamous Danbury Hatters Case, a monumental Supreme Court Decision which effectively outlawed the secondary boycott as a violation of the Sherman Antitrust Act.

In 1902, the United Hatters of North America called for a boycott of the firm of D.E. Loewe in Danbury Connecticut after the latter forcefully implemented an open shop. With the Supreme Court's 1908 decision, the trade union was thought to have violated the rights of individuals through its coordinated and concentrated action. Loewe held that: “the members of a union are liable for the acts of their officers and agents in the same way that a partnership or corporation is.” In other words, the organisation's response had exceeded what could be accomplished by a voluntary assembly of individuals and it should be recognised as an independent legal entity. By forcing trade unionists to reconcile with the actions of their unions, it was held, trade unions would become “what they ought to be, law‐abiding and beneficient organisations entitled to the respect of all” (Ernst 1995, 151).

The decision pleased small employers. Walter Gordon Merritt, son of the Loewe and later legal counsel to the small employers, declared

it is not in one respect, alone, that the case of DE Loewe, et al. versus Martin Lawler et al. is the most extraordinary case that has ever appeared before the courts of this or any other country, it is the first attempt that has ever been made to hold the rank and file of the membership of a large union under full responsibility for the wrongful acts of their officers and agents.

(Loewe 1908, 4).

It enraged labour leaders, who insisted that “it is clearly an unwarranted assumption on the part of the courts or others to place the voluntary associations of the workers in the same category as trusts and corporations” (American Federation of Labor 1908, 263). Looking to their British counterparts, they argued that “if such relief from the onerous conditions brought about by the Taff‐Vale decision of the highest court of Great Britain can be enacted by a monarchical government, there ought to be no hesitancy in conceding it in our own republic” (American Federation of Labor 1908, 263).

Indeed, the Supreme Court decision in Loewe versus Lawler was the legal equivalent to Britain's Taff Vale. And just like Taff Vale, it highlighted the urgency of finding an appropriate organisational category for trade unions. This task would be taken on by the National Civic Federation—an association of business leaders, political activists, and intellectuals born out of the National Conference on Trusts and Combinations held in Chicago in 1899 and led by Republican Party activist Ralph Easley. The NCF was founded in 1900 with the express purpose of improving relations employers and organised labour. Shunning the radical unionism of the IWW, it favoured “respectable” unionism and invited more than 30 labour leaders to hold positions on its advisory committee. In the first decade of the twentieth century, Gompers served as the organisation's first vice‐chairman.

The NCF was typical of the early twentieth century's expert to policy pipeline: it not only gained the support of the Progressive Attorney General Charles Bonaparte, but was the organisational home of multiple members of Roosevelt's cabinet. In 1903, the NCF held a lengthy symposium on the question of trade union incorporation. Employers in the symposium held that organised labour's resistance to incorporation was “an open confession that their methods are illegal and wrong” (Monthly Labor Review 1935, 42). Incorporation would “undoubtedly add to their responsibility,” thereby making it the “quickest way to make them abandon a radical or ill‐considered policy” (National Civic Federation 1903, 2). Labour leaders insisted that “incorporation proceedings would be only a step nearer the danger line which would put the trade organisations at the mercy of the courts” (National Civic Federation 1903, 3). The position of reformers remained consistent. Henry Farnam, founder and president of the AALL, held that incorporation would “add to the feeling of responsibility which the leaders would be governed by” (National Civic Federation 1903, 5).

The symposium therefore confirmed the sentiments of the period: reformers and employers wanted trade unions to legally incorporate, while labour leaders insisted on their status as voluntary associations. In 1907, the NCF would once more attempt a resolution. Its much anticipated 4‐day conference on the question trusts and combinations brought together lawyers, fraternal society leaders, journalists, academics, and trade unionists to reflect on ideal remedies for the state of industrial concentration.

Foremost leaders of the NCF, including Republican Mayor of Brooklyn Seth Low, and economist Jeremiah Jenks, argued that the legal incorporation of all associations and trusts would “rationalise” American industrial relations and facilitate an end to the unplanned use of strikes. This sentiment was reiterated by the overwhelming majority of the conference's speakers. The commission ultimately resolved:

that any combination or organisation of individuals whose acts may control the price, production or traffic in any article of common use or necessity, whether labor, manufactured article or other product, in any state other than wherein it is organized, shall be required to incorporate under the federal laws and thus be amenable to the federal government for any abuse of its power.

(National Civic Federation 1908, 371).

Trade unions were explicitly included in this framework. The newspaper manager Donald C. Seitz suggested, “When one set of men bind themselves together and say that they will only work under certain conditions…they become something apart from the community, and to an extent a menace.” Given their collective pursuit of worker's interests, “the labor union should be compelled to put itself in the attitude of the ancient guild and become a corporation” (National Civic Federation 1908, 412).

The NCF's four day conference would result in the introduction of the Hepburn Bill, a modification on the Sherman Anti‐Trust Act which, among other objectives, sought to adequately deal with the Supreme Court's decision in Loewe versus Lawler. The Bill proposed to grant trade unions immunity for agreements that were submitted to the Bureau and found not to restrain trade unreasonably. Its labour sections legalised boycotts and sympathetic strikes and aimed to withdraw combinations from reach of federal prosecution. These rights were dependent on registration with the Federal Bureau of Corporations and the filing of information on governance, structure, and finances. Effectively, it offered to grant trade unions a legal standing through incorporation (Ernst 1995).

President Theodore Roosevelt initially endorsed the Bill as a cornerstone of the new era of industrial combinations. The Bill also made the right kinds of enemies: testifying before the Committee on the Judiciary, National Association of Manufacturers (NAM) representative James Emery referred to it as “the most dangerous and diabolically ingenious measure yet proposed to congress,” (United States Congress 1913, 3772) while Daniel Davenport of the American anti‐Boycott Association (AABA) warned that if the act were to pass “it would be perfectly lawful for employees of any railroad to refuse to haul the product of any manufacturer having trouble with any of his employees” (United States Congress 1913, 3774).

The Hepburn Bill offered an opportunity for labour leaders to ally with Progressive reformers in the institutionalisation and legitimation of trade union activity. But in their dedication to the corporate form, reformers ultimately alienated the country's trade unions. In his testimony before the Senate Committee on the Judiciary, Gompers insisted that he was “not in accord with the provisions of the Hepburn bill in so far as they apply to the organisation of the wageworkers.” This was, first and foremost, because it was neither “wise [nor] necessary to have the organisations of labour register.” Second, the Bill would force trade unions to “submit their agreements to the Bureau of Corporations or some other Department, to be subject to their approval or disapproval” (Senate Committee on the Judiciary 1908, 166–67). Despite his participation in the conference, he resisted the Bill on the grounds that its exemption from the Sherman Anti‐Trust Act depended on incorporation. Registration with the government, he argued, opened the way for more stringent regulation down the line (Johnson 1961).

With small business interests represented by the AABA, key sections of industry represented by the NAM, and organised labour all turning against the Bill, the coalition in its favour grew fragile. Despite the persistent support of Republican policymakers, economists, and influential social reformers, Roosevelt ultimately concluded that the Bill “would be in the end ruinous politically” (Johnson 1961). Though the Bill received hearings in the Chamber of Congress and a 1909 report by the Senate Judiciary Committee, it ultimately did not pass.

The AFL's break with reformers in the NCF over the Hepburn Bill would sour relationships with insurance advocates in the American Association for Labour Legislation (AALL). Carroll D. Wright, John Commons, and Louis Brandeis were among the organisation's founders and continued to advise it throughout the Progressive Era. They were some of the country's foremost advocates of old age pensions, child labour regulations, and national insurance, collaborating with the AALL leader John Andrews in the drafting and circulation of insurance proposals. In campaigning for trade union incorporation, US liberal reformers shared the interests of their British counterparts: to transform the organizations into respectable and lasting features of the country's political economy. In the US context, however, the means for doing so was complete incorporation—a status that labour leaders across the board could not accept.

The relationship between legal forms and welfare coalitions would once more be demonstrated in 1935. With the passing of the Wagner Act, the US government granted trade unions a legal standing akin to that of the quasi‐corporation. Requiring no registration with the Bureau of Corporations, the act nevertheless granted legal standing to the organizations and their contracts (Fischer 1941). The importance of this legal category to the Progressive coalition that formed is a topic for further study.

## 5 Conclusion

Scholars of the welfare state have debated the conditions for welfare state coalitions. In this article, I have argued that legal forms, and the struggles over them, are crucial in shaping coalitions for welfare reform. During the late nineteenth century, American and British societies experienced a prolonged depression followed by industrial consolidation. In both countries, an internationally coordinated, educated elite advocated for the loosening of laissez‐faire principles and active state intervention in the economy. Hailing from academia, law, business, and government, these reformers held influential positions in party politics and policymaking. In independent studies, conferences, and government appointed commissions, they compiled statistical accounts of poverty, industrial conditions, and living standards in their respective countries. Based on these analyses, they put forward proposals for industrial arbitration, sickness, pensions, unemployment, and accident insurance, public works, and municipal ownership, among many other schemes.

Among the key contribution of Progressives would be a greater recognition for the legitimate functions of trade unions. For the first time, late nineteenth century reports would recognise the utility of trade unions as agents of collective bargaining rather than simply benevolent organisations. In their bargaining efforts, trade unions raised the standard of living for workers, improved working conditions, and thus facilitated more efficient production. If encouraged in this capacity, reformers hoped, they may be less inclined to resort to strikes.

I have sought to demonstrate that the relationship between Progressive reformers and the labour movement was conditioned by the organisational forms that mediated their respective ambitions. Progressive reformers in the UK could increase the respectability and legitimacy of trade union by advocating for the expansion of a “quasi‐corporate” legal form developed for mutual benefit societies. Quasi‐incorporation secured trade union contracts and granted trade unions the right to hold property and be represented in court. It did so, however, through a simple registration process in which the organisation's constitution, by‐laws, and financial accounts were not subject to modification or formulation by government officials. The fight against trade union incorporation enabled a meaningful and durable alliance between reformers and labour leaders—one which would continue into the twentieth century.

Reformers in the US were not faced with a similar option. Since the country's founding, associations of all stripes had easily applied for charters of incorporation. Early general incorporation laws across states facilitated a decentralised mode of governance, whereby local governments closely directed civic life. In advocating for trade union legitimation, Progressives therefore called on the corporate form. But heavy supervision by public authorities was a bargain that AFL leaders were unwilling to strike. In doubling down on voluntarism, they broke with the reformist impulse towards “government by commission” and resolved to preserve their independence from the state—once more delegitimating their collective bargaining function and rendering them reliant on insurance.

In this way, the organisational forms available to political actors guided their positioning on a broader political spectrum. Despite similar policy ambitions, reformers in each country drew on different repertoires in their campaign for trade union legitimation. Inherited organisational infrastructures guided the way their political principles manifested, and thus the sort of political coalitions they formed. Organisational forms thus prove to be an important and understudied force in the coalitional politics of early welfare states.

## Ethics Statement

This work was conducted with publicly available archival sources and is in line with the journal's ethics policy.

## Data Availability

The data that support the findings of this study is drawin from a wide variety of archival sources in the public domain, including: ‐ HathiTrust‐ London Metropolitan University Special Collections‐ UK Parliamentary Archives‐ US Congressional Record‐ ProQuest Newspaper Archives.
